# Pituicytoma Coexisting With Corticotroph Hyperplasia: Literature Review With One Case Report

**DOI:** 10.1097/MD.0000000000003062

**Published:** 2016-03-11

**Authors:** Xiaopeng Guo, Hanhui Fu, Xiangyi Kong, Lu Gao, Wenze Wang, Wenbin Ma, Yong Yao, Renzhi Wang, Bing Xing

**Affiliations:** From the Department of Neurosurgery (XG, HF, XK, LG, WM, YY, RW, BX); Department of Pathology (WW), Peking Union Medical College Hospital, Chinese Academy of Medical Sciences. Beijing, China.

## Abstract

Pituicytoma is a rare, low-grade glial neoplasm that arises in the neurohypophysis or infundibulum and usually presents as pituitary gland enlargement. They are often misdiagnosed as pituitary adenomas. Causes have varied for high serum adrenocorticotropic hormone level reported in a few patients with pituicytoma.

We report a rare case of pituicytoma accompanied by corticotroph hyperplasia—a challenging diagnosis guided by clinical presentations, radiological signs, and biopsy.

We present a case of pituicytoma with corticotroph hyperplasia in a 46-year-old woman with typical Cushing syndrome. Magnetic resonance imaging revealed a lesion in the sellar area with equal T1 and T2 signals and marked homogeneous enhancement. We present detailed analysis of the patient's disease course and review pertinent literature. Written informed consent was obtained from the patient for publication of this case report and any accompanying images. A copy of the written consent is available for review by the Editor of this journal. Because of this, there is no need to conduct special ethic review and the ethical approval is not necessary.

The patient underwent a surgical exploration and tumor resection through a trans-sphenoidal approach. Pathologic results revealed pituicytoma and corticotroph hyperplasia. As adrenocorticotropic hormone and cortisol levels did not decrease to normal, the patient received radiotherapy and recovered uneventfully. No recurrence was found over 8 years of follow-up.

Pituicytoma is a rare type of sellar tumor. Pituicytomas in patients with Cushing syndrome are rarer still. To our knowledge, this is the first report of Cushing syndrome caused by corticotroph hyperplasia in a pituicytoma patient.

## INTRODUCTION

According to current World Health Organization (WHO) classifications for central nervous system (CNS) tumors, pituicytoma is now reserved for low-grade glial neoplasms (grade I) derived from neurohypophyseal pituicytes, a modified glial cell found in the posterior pituitary gland and infundibulum, which is distinct from pilocytic astrocytomas.^1^ In 2000, Brat et al^2^ proposed a modern depiction of the histopathological features of pituicytomas. Up to now, there have been only less than 60 cases reported to match this depiction. Pituicytomas can lead to local tumor-mass effects such as optic chiasm compressive symptoms, pituitary hormone disorders secondary to pituitary gland compression or pituitary stalk compression, and cavernous sinus syndrome caused by possible lateral invasions. Honegger once reported a pituicytoma patient who presented with Cushing syndrome caused by an adrenocorticotropic hormone (ACTH) microadenoma.^3^ The golden criteria of diagnosis for pituicytomas are histopathological and immunohistochemistry (IHC) examinations, since clinical manifestations and radiological features both are nonspecific.^4^

In this study, we present a case of a 46-year-old woman suffering from Cushing syndrome. Magnetic resonance imaging (MRI) showed a sellar lesion with equal T1 and T2 signals and marked homogeneous enhancement. We originally supposed the tumor to be an ACTH pituitary adenoma and performed a trans-sphenoidal surgery, but histopathological examination corrected the diagnosis to pituicytoma coexisting with corticotroph cell hyperplasia. To our knowledge, this is the first reported case of pituicytoma with corticotroph hyperplasia presenting in this manner. The relevant literature is reviewed and discussed.

## CASE REPORT

The patient is a 46-year-old Chinese female who developed symptoms and signs of Cushing syndrome, including supraclavicular and dorsocervical fat pads, a moon-shaped face with increased frequency of acne, thin extremities, severe fatigue and fairly profound muscle weakness, a tendency to bruise easily, memory dysfunction, anxiety, and insomnia for around 2 years before her visit at our hospital. Over the half-year period before diagnosis, she developed rapidly progressive opsomenorrhea and noted enlargement of her abdomen with a weight gain from 65 to 80 kg. She had also complained of different degrees of headache, which was mainly focused on the forehead, and could be alleviated by rest or analgesics. She denied polyuria and nocturnal enuresis, and had no special family history or personal history. Upon physical examination, the patient's BMI was 38.5 kg/m^2^ (obese) and her blood pressure was 147/105 mm Hg in despite of undergoing a daily enalapril maleate therapy. Her muscle strength of lower extremities was grade 4/5. No gross visual field defects were detected. Sensation function and tendon reflexes were almost normal. Pathological signs and ataxia were absent.

Table 1 shows the details of her pituitary gland studies, showing the serum ACTH and 24-hour urinary-free cortisol were much higher than the normal. Serum cortisol could not be suppressed in an overnight 1 mg dexamethasone suppression testing; in response to a 48-hour 2 mg dexamethasone suppression testing, however, serum cortisol decreased by 62%, suggesting a diagnosis of Cushing disease, characterized by increased secretion of ACTH from the anterior pituitary. Liver and kidney function tests, complete blood count, and immunological and rheumatic indicators were all within the normal range.

**TABLE 1:**
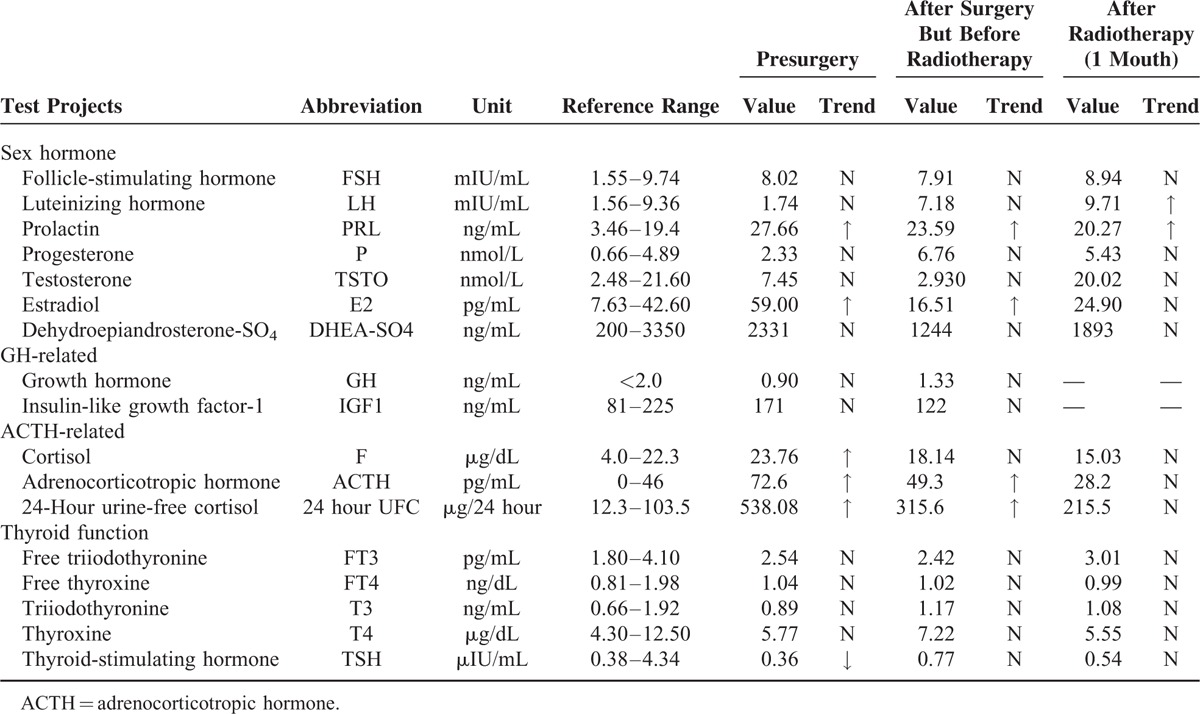
Results of Endocrine Studies for the Pituitary Gland Before and After Surgery

**FIGURE 1:**
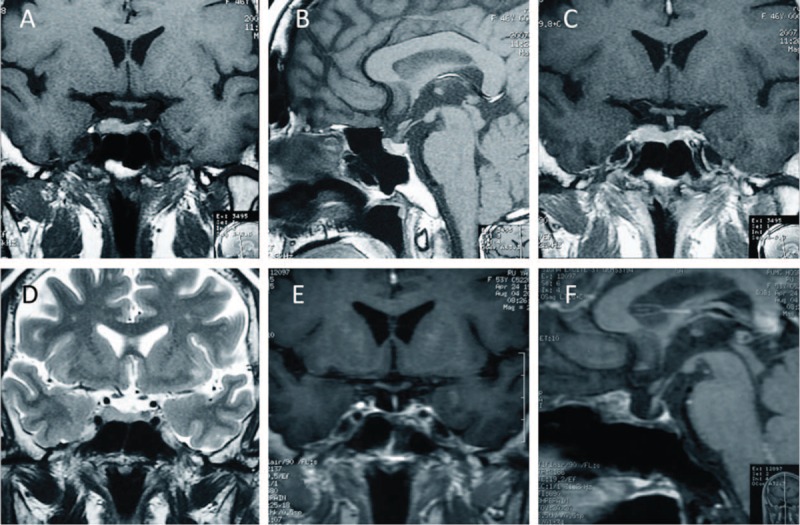
Magnetic resonance imaging (MRI) shows an abnormal enlarged parenchymal lesion in the sellar region.

An MRI revealed an abnormal enlarged parenchymal lesion in the sellar region (especially in the left inferior pituitary gland), measuring about 15 mm × 10 mm × 7 mm (Figure 1). The lesion presented with equal T1 and T2 signals benchmarked against the gray matter, and showed marked homogeneous enhancement following intravenous (i.v.) administration of gadolinium-diethylenetriamine penta-acetic acid (Gd-DTPA). The MRI also showed a thickened pituitary stalk with homogeneous signal intensity, but with no mass effect on the optic chiasm. For the posterior pituitary, T1WI showed a relatively normal short signal. In line with these images, a pituitary adenoma, the most common type of sellar lesion, was initially considered. To exclude possible ectopic ACTH syndrome, computed tomography (CT) scans of the thorax, abdomen, and pelvis were also performed and were negative for any masses. As the MRI appearance is clear, to minimize medical invasive manipulations, we did not perform the inferior petrosal sinus sampling (IPSS) before surgery.

A microscopic endonasal transsphenoidal approach was taken to provide the greatest access to the sellar mass with the lowest risk to the patient. The tumor, mainly within the sella turcica, was reddish and soft, and bled easily under an intraoperative view. It was grossly removed by the naked eye using suction, tumor forceps, and ring curettes. In an intraoperative histological examination with frozen section, the tissue was interpreted as an adenoma of the pituitary and adjacent neurohypophysis tissue. No cerebrospinal fluid was observed. The sellar floor was reconstructed with a piece of nasal septal cartilage. The nasal cavity and nasopharynx were suctioned, and hemostatic sponges were inserted.

**FIGURE 2:**
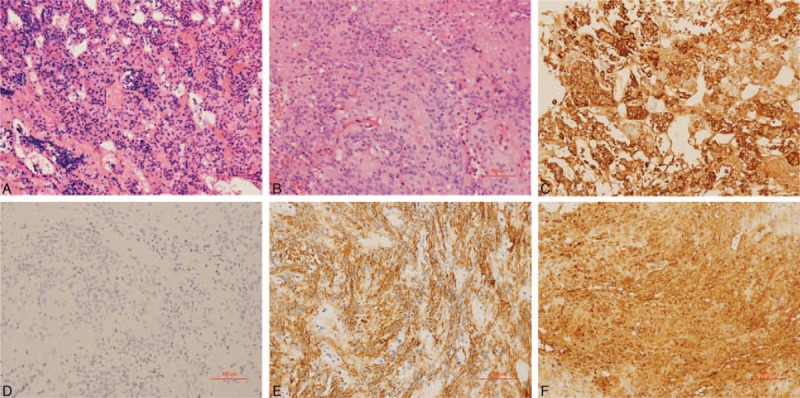
Histopathological images. Cells were short-spindle and ovoid-shaped with vacuoles in the nuclei. No nuclear atypia or mitotic activity was present (A and B; H-E stain). Immunohistochemistry of adenohypophysis tissue around the tumor showed corticotroph hyperplasia (C, ACTH ++). Immunohistochemistry also indicated strong staining for GFAP (D), NF (E), and S-100 protein (F). ACTH = adrenocorticotropic hormone, GFAP = glial fibrillary acidic protein, NF = nuclear factor.

Quite unexpectedly, the eventual postsurgical histological analysis corrected the initial diagnosis to pituicytoma and adjacent ACTH-secreting pituitary hyperplasia (Figure 2). The tumor was composed of stretched dipolar spindle-shaped cells whose cytoplasm was pink-stained. There were no nuclear abnormalities and pathologic mitosis. Their IHC profile showed diffuse positivity for glial fibrillary acidic protein (GFAP), nuclear factor (NF), and S-100; and negativity for epithelial membrane antigen (EMA), CD-34, and synaptophysin with 1% monoclonal antibody (MIB)-1 proliferative index. These findings are characteristic of the diagnosis of a pituicytoma. At its periphery, IHC of the pituitary tissue showed corticotroph hyperplasia with many acinar-like structures; the pituitary hormone ACTH was strongly positive. At this point, a pituicytoma coexisting with ACTH-secreting pituitary hyperplasia was finally diagnosed.

However, although postoperative MRI showed the tumor was generally removed, the ACTH and cortisol levels did not decrease to normal 2 weeks postoperatively (Table 1), suggesting an insidious residual. Another 2 weeks later, after a multidisciplinary discussion, the patient was started on radiotherapy (Dt56Gy, 28 times over 2 months) and was then rechecked on pituitary hormone levels; both serum cortisol and ACTH had returned to normal levels (Table 1). Meanwhile, the clinical symptoms and signs of Cushing syndrome almost disappeared. The patient has been followed up for nearly 8 years now, and the disease seems to be in remission with no recurrence noted.

## DISCUSSION

Pituicytoma was first described by Liss in 1958. It is well-recognized, but exceptionally rare, and commonly appears as a solid, well-circumscribed, noninfiltrative lesion that occupies the sella turcica or the suprasellar region.^5^ According to a review of a handful of case reports by Pirayesh, pituicytomas are slightly more common in men (56%) and tend to occur during the middle decades of life (mean age: 50 years; range: 23–83 years).^6^ Pituicytomas are hypothesized to arise from the posterior pituitary lobe or in the lower portion of the stalk, and extend gradually upwards; they are believed to originate from the neoplastic transformation of pituicytes, which are glial cells that occupy perivascular zones of the neurohypophysis, which regulates hypothalamic hormone release.^6^ Some authors propose that pituicytomas originate from folliculostellate cells of the adenohypophysis for completeness sake, despite a lack of convincing evidence. Five different types of pituicytes have been identified histologically: major cells, dark cells, oncocytes, ependymal cells, and granular cells, with each cell type believed to give rise to a distinct tumor type. The first 2 are the most common.^7^ In the 2007 WHO classifications for central nervous system tumors, pituicytomas are classified as rare, solid, grade I glial tumors.^8^

Generally, the location of the lesions determines the clinical features of pituicytomas.^9^ Those located in the sella region usually show symptoms similar to pituitary adenomas. The common endocrinological abnormalities include hyperprolactinemia, hypopituitarism, male hypogonadism, and low testosterone. Lateral expansion of the mass into the cavernous sinus could cause compression against the oculomotor nerve, trochlear nerve, or abducent nerve, leading to symptoms like oculomotor paralysis. Suprasellar lesions often present with visual disturbances due to compression of the optic nerve or chiasm. Although the neurohypophysis is associated with the storage and release of antidiuretic hormone, and is believed to be the origin of pituicytomas, only 5% of all reported cases presented with central diabetes insipidus (DI), for reasons that are unclear.^10^ In our case, the patient also denied any symptoms of DI; her primary clinical presentation was Cushing syndrome.

High serum ACTH levels were noted in only 5 previous cases, with our case as the sixth one (Table 2).^3,11–14^ Notably, the reasons for high ACTH levels vary among these cases, including a pure mass effect that might disturb the hormone secretion, and coexistence of a microadenoma neglected originally. Whether pituicytomas are associated with other endocrine pathologies is unclear, due to the rarity of described cases. In the present case, pituitary corticotroph hyperplasia confirmed by histopathological and immunohistochemical tests ultimately gave the explanation. To our knowledge, this is the first reported case of a pituicytoma coexisting with corticotroph hyperplasia. Corticotroph hyperplasia as a cause of Cushing disease, although uncommon, has been described in the adult population and has been shown to precede adenoma development, or even to coexist with adenomas.^15^ However, this seems to be exceedingly rare in patients with a pituicytoma. In addition, Cushing disease is almost invariably due to corticotroph adenomas rather than pituicytomas.^16^ Therefore, the present unique case has certain educational significance; whether pituicytes can secrete ACTH may warrant some investigation. In fact (as mentioned above), their histogenesis is still debated, with recent reports suggesting that pituicytomas arise from the folliculo-stellate cells of the adenohypophysis, which are nonendocrine spindled cells that express S-100 and Bcl-2. Of note, to minimize extra invasive manipulation, it is really a pity in this case that IPSS was not done before surgery. The IPSS test could not only help to determine whether the elevated ACTH comes from peripheral or central, but also could help to determine whether the source of ACTH tend to be left or right. This is helpful for exploratory during surgery and to remove the lesion completely. In certain cases, especially those where the ACTH source is not clear before surgery, IPSS is highly recommended.

**TABLE 2:**
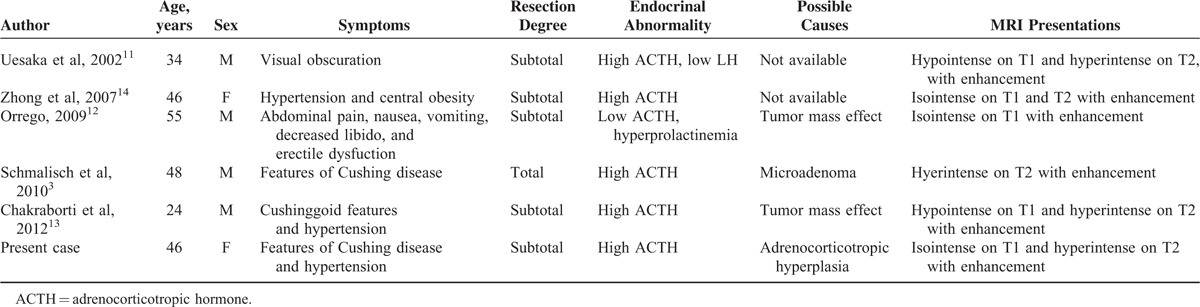
Clinical Review of 5 Cases Published of Pituicytomas With ACTH Disturbance

Differentiating pituicytomas from common sellar region lesions such as pituitary adenoma or meningioma is difficult if based only on their MRI appearance, partly because their imaging findings are not specific enough. In fact, most reported pituicytomas were misdiagnosed before surgery.^17^ Sometimes, they can exhibit sellar enlargement and bony remodeling. Most reported MRI findings describe a solid, homogenous mass, iso-intense on T1-weighted images and hyperintense on T2-weighted images, with homogenous contrast enhancement, closely mimicking pituitary adenoma or meningioma.^9^ Heterogeneous enhancement, calcification, and cystic changes were rarely seen.^17^ In our case, the lesion presented with equal T1 and T2 signals, which accords with previous reports.

As the neuroimaging features of pituicytomas are nonspecific, its diagnosis relies on pathological findings.^4^ Grossly, pituicytomas are discrete, solid, well-circumscribed tumors with a firm rubbery texture; their size may reach several centimeters. Histopathologically, pituicytomas are characterized by a compact architecture of elongated bipolar spindle cells arranged in interlacing fascicles or a storiform pattern.^18^ Kwon and Suh^19^ once presented an unusual pituicytoma case with a vague perivascular pseudorosette histological pattern, large multinucleate pleomorphic cells, and scattered Herring bodies, which implies origination from ependymal pituicytes. Generally, pituicytomas are noninfiltrative and immunoreactive for vimentin, S-100 protein, and GFAP (often focally), with occasional cytoplasmic EMA positivity. Mitoses are extremely rare; MIB-1 labeling indices are low at 0.5% to 3%.^3,20^

The best treatment of pituicytoma is gross total resection via a trans-sphenoidal approach, both of which release the mass effect and provide histopathological specimens, which are especially valuable in cases with atypical clinical and radiological presentations and unconfirmed diagnoses.^6^ As pituicytoma is a slow-growing and rare low-grade glial neoplasm, no recurrence has been reported after gross total resection, although follow-up periods have varied. However, the highly vascular nature of the tumor and its potential for infiltration can make total resection difficult; recurrence is therefore a possibility.^21^ Under this circumstance, radiotherapy to control residual tumor and increase survival is recommended, although management is uncertain due to the rarity of this tumor.^13^ In our case, the ACTH and cortisol levels did not decrease to normal by 2 weeks after resection, although postoperative MRI showed a satisfying tumor removal. As preoperative examinations indicated that ectopic ACTH syndrome was unlikely, these results implied an insidious residual tumor, possibly with corticotroph hyperplasia. The patient underwent radiotherapy and her ACTH and cortisol levels eventually decreased to normal levels. Now, after 8 years of outpatient follow-up care, the patient's postoperative condition has remained uneventful. To our knowledge, this is the longest reported follow-up period for pituicytoma.

## CONCLUSIONS

We report here a rare case of pituicytoma in the neurohypophysis, coexisting with ACTH-secreting pituitary hyperplasia in adenohypophysis—a combination which has not been previously reported. Pituicytomas are not easily differentiated from other common sellar region lesions; accurate diagnosis relies on pathological examination. Corticotroph hyperplasia is an uncommon cause for Cushing syndrome, but should be considered for patients with both Cushing syndrome and pituicytoma. Postoperative radiotherapy is a possible treatment for such cases where residual tumors are suspected.
